# Liraglutide in the treatment of heart failure: insight from FIGHT and LIVE

**DOI:** 10.1186/s12933-020-01088-3

**Published:** 2020-07-06

**Authors:** Bo Liang, Ning Gu

**Affiliations:** 1 Nanjing University of Chinese Medicine, Nanjing, China; 2 Nanjing Hospital of Chinese Medicine Affiliated to Nanjing University of Chinese Medicine, Nanjing, China

**Keywords:** Glucagon-like peptide-1, Liraglutide, Heart failure, FIGHT, LIVE

## Abstract

There are many glucose-lowering agents used in patients with heart failure, showing mixed results, this study was conducted to determine the effect of liraglutide, a glucagon-like peptide-1 analogue, on the treatment of patients with heart failure. Patients from the FIGHT and LIVE trials were included, all overlapped data were summarized and described. No significant changes from baseline in left ventricular ejection fraction, N-terminal pro-B-type natriuretic peptide, hemoglobin A1c, heart rate, left ventricular end-systolic volume index, left ventricular end-diastolic volume index, and 6 min walk test were observed in FIGHT. In LIVE, liraglutide significantly decreased hemoglobin A1c and inceased 6 min walk test and increased heart rate and serious cardiac adverse events, and there were no statistical differences in left ventricular ejection fraction, N-terminal pro-B-type natriuretic peptide, left ventricular end-systolic volume index, and left ventricular end-diastolic volume index. In this study, we found that there is not enough reason to support the use of liraglutide in patients with heart failure, and importantly, the safety of liraglutide in this particular population remains uncertain. Enhanced recognition the risks and benefits of liraglutide would help guide therapeutic decisions in patients with heart failure.

Heart failure (HF) affects at least 26 million people worldwide, and the prevalence is increasing year by year. HF patients experience a high burden of symptoms and functional limitations, and morbidity and mortality remain high despite successful therapies [1]. Type 2 diabetes mellitus (T2DM), the most frequent subtype of diabetes, is an expanding global health problem [2], and in the past decades, the global prevalence has increased by 30%, and now about 463 million people are affected. Both HF and T2DM independently increase the morbidity of another disease and associated with considerable mortality [3, 4]. There are many glucose-lowering agents used in patients with HF [5], however, the results are mixed [6]. Here, we critically and systematically explored the effect of liraglutide, a glucagon-like peptide-1 (GLP-1) analogue, on the treatment of patients with HF.

We included all perticipants from the Functional Impact of GLP-1 for Heart Failure Treatmen (FIGHT) [7] and Effect of Liraglutide on Left Ventricular Function in Stable Chronic Heart Failure Patients with and without Diabetes (LIVE) [8] trials, which are multicentre, double-blind, randomised, placebo-controlled clinical trial. Two reviewers extracted data independently and crossed check. Only data indicators from both trials can be used for subsequent analysis. All the results were descriptive analysis.

In those trials, 541 perticipants from 24 sites in the United States and 4 Danish Centres were included. The baseline of two trials was basically similar, regardless of general data, functional measures, physical examination, medical history, laboratory measures, echocardiographic data, and HF medications (Table 1). The outcomes of FIGHT and LIVE are not exactly the same, and we choose the coincident indicators for analysis. In FIGHT, liraglutide decreased left ventricular ejection fraction, N-terminal pro-B-type natriuretic peptide, hemoglobin A1c and heart rate, and increased left ventricular end-systolic volume index, left ventricular end-diastolic volume index, and 6 min walk test, compared with placebo. However, there was no statistical difference. In LIVE, liraglutide significantly decreased hemoglobin A1c (*P* < 0.0001) and inceased 6 min walk test (*P* = 0.04) and heart rate (*P* < 0.0001), compared with placebo. There were no statistical differences in the influence of the abovementioned outcomes that coincided with FIGHT (Table 1).

**Table 1:** The details of FIGHT and LIVE trials

	FIGHT		LIVE	
	Liraglutide (N = 154)	Placebo (N = 146)	Liraglutide (N = 122)	Placebo (N = 119)
Genaral data				
Age	62 (52–68)	61 (51–67)	65 ± 9.2	65 ± 10.7
Male	123 (80%)	113 (77%)	109 (89%)	106 (89%)
BMI (kg/m^2^)	31 (26–36)	33 (25–38)	28.0 ± 3.8	29.8 ± 4.6
Functional measures				
6MWT (m)	234 (143–313)	212 (141–311)	453 ± 104	441 ± 122
NYHA				
I	4 (3%)	8 (5%)	36 (30%)	35 (29%)
II	49 (32%)	36 (25%)	65 (53%)	64 (54%)
III	93 (60%)	96 (66%)	17 (14%)	16 (13%)
IV	8 (5%)	6 (4%)	4 (3%)	4 (3%)
Physical examination				
SBP	108 (99–120)	108 (99–118)	128 ± 20	127 ± 18
HR	75 (68–85)	76 (68–88)	67 ± 10	67 ± 11
Medical history				
T2DM	91 (59%)	87 (60%)	39 (32%)	35 (29%)
AF	74 (48%)	70 (48%)	34 (28%)	34 (29%)
Ischemic heart disease	133 (86%)	113 (77%)	72 (59%)	73 (61%)
Laboratory measures				
NT-proBNP (pg/mL)	1936 (1075–4231)	2083 (1020–4333)	413 (208–926)	388 (153–880)
HbA1c (%)	6.6 (6.6–7.6)	6.7 (5.9–7.9)	5.9 ± 0.7	6.0 ± 0.8
Echocardiographic data				
LVEF (%)	25 (20–33)	25 (19–32)	33.7 ± 7.6	35.4 ± 9.4
LVESV (mL)	104 (78–130)	100 (80–1330	111 ± 60	109 ± 78
LVEDV (mL)	140 (112–173)	137 (115–174)	163 ± 71	165 ± 111
E/e'	22 (17–28)	23 (18–30)	12.6 ± 6.0	11.7 ± 5.5
Heart failure medications				
ACEI/ARB	112 (73%)	104 (71%)	118 (97%)	115 (97%)
β-Blocker	143 (93%)	139 (95%)	113 (93%)	108 (91%)
Aldosterone recepter antagonist	88 (57%)	89 (61%)	59 (48%)	54 (45%)
Loop diuretic	151 (98%)	146 (100%)	90 (74%)	90 (76%)
Lipid-lowering agent	110 (71%)	110 (75%)	96 (79%)	92 (77%)
Treatmen effect				
LVEF	− 0.1 (− 2.3–2.1)		− 0.8 (− 2.1–0.5)	
LVESV	5 (− 2.6–12.7)		2.6 (− 1.2–6.4)	
LVEDV	6.7 (− 2.6–16)		3.4 (− 2.3–9.2)	
6MWT	5 (− 29–39)		24 (2–47)	
NT-proBNP (pg/mL)	− 155 (− 1368–1058)		− 140 (− 317–37)	
HbA1c (%)	− 0.33 (− 0.67–0)		− 0.4 (− 0.5– − 0.3)	
HR	− 1.6 (− 4.8–1.6)		7 (5–9)	

*FIGHT* Functional Impact of GLP-1 for Heart Failure Treatmen, *LIVE* Effect of Liraglutide on Left Ventricular Function in Stable Chronic Heart Failure Patients with and without Diabetes, *BMI* body mass index, *6MWT* 6-min walk test, *NYHA* New York Heart Association, *SBP* systolic blood pressure, *HR* heart rate, *T2DM* type 2 diabetes mellitus, *AF* atrial fibrillation, *NT-proBNP* N-terminal pro-B-type natriuretic peptide, *HbA1* chemoglobin A1c, *LVEF* left ventricular ejection fraction, *LVESV* left ventricular end-systolic volume index, *LVEDV* left ventricular end-diastolic volume index, *E/e'* ratio of early mitral inflow velocity to early diastolic medial mitral annular velocity, *ACEI* angiotensin-converting enzyme inhibitor, *ARB* angiotensin receptor blocker

In LEADER, the rate of the primary composite outcome in the time-to-event analysis (the first occurrence of death from cardiovascular causes, nonfatal myocardial infarction or nonfatal stroke) among patients with T2DM and high cardiovascular risk was lower with liraglutide than with placebo, whereas the number of hospitaion for HF was reduced non-significantly in the active arm [9]. The encouraging results of this study may only indicate the potential strong benefits of liraglutide for diabetic patients with high cardiovascular risk. In our study, we specifically included patients with demonstrated HF, not with high cardiovascular risk, to evaluate the effectiveness and safety of liraglutide, and we concluded that liraglutide may be helpful for patients with HF. After careful investigation, we found that the results of FIGHT and LIVE were not identical. The reasons may be various. Firstly, the prescription of liraglutide is different. Liraglutide dosage uptitration as tolerated every 14 days from 0.6 to 1.2 to 1.8 mg/d during the first 30 days of the FIGHT trial, whereas subcutaneous injectable liraglutide was introduced at a dose of 0.6 mg/day, which was increased to 1.2 mg/day after 1 week and to 1. 8 mg/day thereafter in the LIVE trial. Although a dose increase could be postponed depending on the patient's tolerance to the trial product or reduced at any time during the trial if required, different administration of liraglutide may also have an impact on the results. For later trials, we may need a unified way of liraglutide dosing. The second may be recommended because it is consistent with LEADER [9, 10]. Secondly, though HF with left ventricular ejection fraction < 45% was included, it was clear that the degree of HF in FIGHT was more severe than that in LIVE, because it was dominated by New York Heart Association Class II and III (90%). Additionally, two studies were conducted in different country, which may result in excursion in race. Finally, results from LIVE indicated that treatment with liraglutide was associated with an increase in heart rate and more serious cardiac adverse events. More data on the safety of liraglutide in patients with HF are needed.

In this study, we found that there is not enough reason to support the use of liraglutide in patients with HF, which is consistent with the latest updated meta-analysis of 43 randomized controlled trials [11]. Another a network meta-analysis of 171,253 participants from 91 randomized controlled trials also showed that GLP-1 analogue was significantly inferior to sodium-glucose co-transporters 2 inhibitors in terms of HF risk [12]. Among patients with HF and T2DM, after adjusting for baseline characteristics and disease risk factors, the use of GLP-1 analogue was associated with higher risk of HF hospitalization compared to dipeptidyl peptidase-4 inhibitors, though the association was not statistically significant [13]. In the spontaneously developed HF model of J2N-k hamsters, liraglutide exerted further deteriorated cardiac function with overt fibrosis and cardiac enlargement [14]. However, clinical evidences showed that liraglutide reduced early left ventricular diastolic filling and left ventricular filling pressure, thereby unloading the left ventricle [15, 16]. Renal dysfunction is often associated with HF. Interestingly, liraglutide was not associated with excess risk of worsening renal function compared with placebo [17], result from post hoc analysis of FIGHT, supporting the relative renal safety profile of liraglutide among patients with HF. Additionaly, GLP-1 analogue reveals cardiovascular and renal protective effects, although this effect is not as evident as sodium-glucose co-transporters 2 inhibitor [18, 19].

In summary, the results of the body of existing evidence do not support the use of liraglutide in HF. Importantly, the safety of liraglutide in this particular population remains uncertain. Therefore, further studies are needed to assess the risks and benefits of liraglutide in patients with HF.

## Data Availability

Not applicable.

## Abbreviations

FIGHT: Functional Impact of GLP-1 for Heart Failure Treatmen
GLP-1: Glucagon-like peptide-1
HF: Heart failure
LIVE: Effect of Liraglutide on Left Ventricular Function in Stable Chronic Heart Failure Patients with and without Diabetes
T2DM: Type 2 diabetes mellitus
